# Perforation of Appendix Vermaformis; Death in Thirty-Seven Hours

**Published:** 1876-02

**Authors:** Chas. Warrington Earle

**Affiliations:** Chicago


### PERFORATION OF THE APPENDIX VERMAFORMIS; DEATH IN THIRTY-SEVEN HOURS.

By CHAS. WARRINGTON EARLE, M.D., Chicago.

Thomas Harbridge was a healthy, robust, and temperate man; of English origin; aged 29; a contractor by occupation. His health up to the time I was called, had been such that he had not required the services of a physician for fifteen years.

I was called to see this gentleman, March 9th, 1873, and found that the day previous he had eaten very heartily of veal, indeed had made his dinner meal almost entirely of it.

He felt very uncomfortable for three or four hours, and finally was seized with severe pain in the region of the umbilicus. He was habitually constipated and the evening before, as was quite usual for him, had taken a dose of some kind of cathartic pills, which had, as yet, caused no evacuation of the bowels.

I ordered a laxative sufficient to move his bowels ; hot fomentations to the abdomen, and anodynes, after the action of the cathartic, in such quantity as would give him ease.

I diagnosticated the case as indigestive colic, and expressed my belief to my patient that his sickness would be of short duration—that after a complete evacuation of the bowels he would be comparatively free from pain. Upon taking my departure I requested to be notified if he was not better the following day.

I was recalled March 12th, and informed that after the action of the medicine which had been ordered three days before, the patient had been greatly relieved, but at present he was suffering with a diarrhœa and a slight diffused soreness in the abdomen. A more careful examination showed a tongue covered with a whitish fur, red at the end, and dry ; a pulse at 120 ; temperature in the axillæ, $101\frac{1}{5}$ ; slight tenderness in the right iliac fossa. He was ordered the turpentine emulsion, and an easily digested diet. In the evening his pulse was 120 ; temperature, 102.

March 13. Patient better in every respect. His fever had subsided ; the diarrhœa had ceased ; the appearance of the tongue is improved. He was given an acid tonic (the acid arom. sulph., with some carminative), with instruction to resume the emulsion if his diarrhœa returned.

March 14. Visited Mr. H. at 9.30 A. M., and found him sitting in a chair and feeling very comfortable. He had eaten a fair breakfast and expressed himself as "all right, only a little sore from the pain, and weak." His pulse was at 90 ; I counted it at least twice. He complained of *no local pain whatever*. There was at this time no particular soreness even in the right iliac fossa, indeed, the slight pain that he experienced, was more like a diffused lameness, from the severe spasmodic pains of the preceding days. He was to continue the tonic; the light and easily digested diet; and was cautioned to keep quiet.

One hour and a half later I received an urgent call to revisit Mr. H. I obeyed the summons as speedily as possible and found my patient in collapse—covered with a cold, clammy perspiration—pale and nearly pulseless. I learned that a short time after my morning visit, he felt well enough to walk down stairs, and while standing conversing with one of his workmen was suddenly seized with a most violent pain in the abdomen, and was in a few minutes in collapse.

I administered diffusible stimulants with anodynes; applied heat to the extremities, and fomentation to the abdomen, and by one o'clock P. M. his pulse was somewhat better, yet the pain was severe. At three o'clock P. M. his pulse was not as good; his face was congested; extremities still cold; countenance hypocratic; indeed he was still in collapse, and nothing that I could do was rallying him.

There was no doubt in my mind, at this time, as to the nature of the difficulty we had to combat. It was evident that some foreign substance had been emptied into the peritoneal cavity. What it was I could not tell. The previous history of the case would hardly justify any definite opinion. I immediately asked for, and it was decided to have, a general consultation.

That evening Drs. W. G. Dyas, T. D. Fitch and A. H. Foster visited the patient with me. After a careful consideration of all the symptoms present in the case, it was decided that the collapse from which our patient was suffering, and which already foreshadowed a most serious result, was peritonitis, caused by one of three things, to wit: perforation of the bowel from some ulcerative process—rupture of a blood vessel—or extravasated blood from minute openings.*

* Broussais and some other authorities speak of the induction of peritonitis by the exudation of blood into the abdominal cavity, without solution of continuity in any of the blood vessels. * * * * *

It was also agreed that opiates and stimulants by mouth, and fluid nutriment by rectum, would give us the only hope of successfully combating the difficulty, and restoring our patient to his anxious friends.

The morning of the 15th, Dr. Fitch saw the case with me. There was certainly no change for the better. His pulse, if any difference could be detected, indicated a more serious condition. There was tympanitis over a large part of the abdomen, and dullness in the *left iliac fossa.* No symptoms of general effusion were present. Early in the day the patient was seen by Dr. Tree, a gentleman of more than forty years practice, the family physician to the parents of Mr. Harbridge. As far as I have learned, he coincided with the opinions advanced by the medical gentlemen in attendance, in both diagnosis and treatment, and, at his suggestion, the ung. hydrargyri was thoroughly applied to the abdomen. All our efforts were in vain, however, and death took place thirty-seven hours after his sudden attack.

A post mortem examination was made about twelve hours after death, at which were present several physicians and a number of relatives and friends of the deceased.

The cadaveric rigidity was not well marked, indeed we would not expect it; for in the case of persons killed suddenly in full health, or dying after a very brief sickness, the rigidity of the muscles may not take place for thirty hours. The body was well nourished, there being from one to two inches of adipose tissue between the integument and abdominal muscles. Both the peritoneum and intestines showed the effect of general inflammation. In the abdominal cavity was found a large

According to Broussais, the pulse is at first full, soon becoming soft and compressible, the pain very acute, often intermittent, the extremities are cold and convulsions quickly close the scene. Laennec also spoke of hæmorrhagic exudations from serous membranes, and Rokitansky attributes such tendency to certain blood changes, such as the tubercular and alcoholic cachexiæ, the scorbutic constitution, etc., etc.—*Reynolds' System of Medicine, Vol. III.*

amount of albuminous exudation and a small amount of serum.

As the intestines were partially removed from their place, we found, in about the central part of the abdominal cavity (a little to the right) an oval, bean-shaped substance, resembling very closely a peanut, indeed several gentlemen remarked at once the resemblance I have named.* As we proceeded to examine the right iliac fossa, evidence of pus was found, and when the cæcum was withdrawn, the secret of the trouble was plainly manifest. The appendix was inflamed throughout its entire length, and perforation had taken place at three points. The largest opening, and the one from which the concretion mentioned above probably escaped, was about one-half inch from the bowel, the two other openings were near the pointed extremity. Within the tube, and between the openings mentioned, were found four other concretions, similar to the one which had escaped into the peritoneal cavity.

There was a large amount of pus in and around the appendix, and the evidences of a severe and destructive inflammation were very marked.

* My patient had been a great lover of peanuts, and generally had a supply with him, eating them while at his work.

